# Targeting Dormant Bacilli to Fight Tuberculosis

**DOI:** 10.4084/MJHID.2013.072

**Published:** 2013-11-19

**Authors:** Lanfranco Fattorini, Giovanni Piccaro, Alessandro Mustazzolu, Federico Giannoni

**Affiliations:** 1 Dipartimento di Malattie Infettive, Parassitarie e Immunomediate, Istituto Superiore di Sanità, Roma

## Abstract

Tuberculosis (TB) is an infectious disease caused by *Mycobacterium tuberculosis* (Mtb), which kills about 2 million people annually. Furthermore, 2 billion people worldwide are latently infected with this organism, with 10% of them reactivating to active TB due to re-growth of nonreplicating (dormant) Mtb residing in their tissues. Because of the huge reservoir of latent TB it is important to find novel drugs/drug combinations killing dormant bacilli (microaerophiles, anaerobes and drug-tolerant persisters) surviving for decades in a wide spectrum of granulomatous lesions in the lungs of TB patients. Antibiotic treatment of drug-susceptible TB requires administration of isoniazid, rifampin, pyrazinamide, ethambutol for 2 months, followed by isoniazid and rifampin for 4 months. To avoid reactivation of dormant Mtb to active pulmonary TB, up to 9 months of treatment with isoniazid is required. Therefore, a strategy to eliminate dormant bacilli needs to be developed to shorten therapy of active and latent TB and reduce the reservoir of people with latent TB. Finding drugs with high rate of penetration into the caseous granulomas and understanding the biology of dormant bacilli and in particular of persister cells, phenotypically resistant to antibiotics, will be essential to eradicate Mtb from humans. In recent years unprecedented efforts have been done in TB drug discovery, aimed at identifying novel drugs and drug combinations killing both actively replicating and nonreplicating Mtb *in vitro*, in animal models and in clinical trials in humans.

## Introduction

Tuberculosis (TB) is an infectious disease caused by *Mycobacterium tuberculosis* (Mtb), a microorganism firstly described by Robert Koch in 1882. After more than one century the disease has not yet been eradicated and in 2011 the World Health Organization (WHO) estimated an incidence of 8.7 million new cases (13% co-infected with HIV) and 1.4 million deaths from TB.^1^

Active TB is quite well controlled by antibiotic treatment, but therapy lasts 6 months including 2 months of isoniazid (INH), rifampin (RIF), pyrazinamide (PZA), ethambutol (EMB), and 4 months of INH and RIF.^2^–^3^ The WHO makes great efforts to facilitate drug treatment in Africa, Asia and in the former Soviet Union by the so-called directly observed therapy (DOT) ensuring the patients actually take medications. Indeed, poor adherence to therapy is one of the reasons why particularly in low-income countries is increasing the number of TB patients harboring multidrug-resistant (MDR) Mtb strains (*i.e.* resistant to at least INH and RIF, the two most powerful drugs) and extensively drug-resistant (XDR) strains [*i.e.* MDR strains resistant to any fluoroquinolone and to at least one injectable second-line drug (amikacin, kanamycin, capreomycin)].^4^ In many industrialized countries the majority of MDR/XDR cases occurs in foreign-born people emigrated from regions where drug-resistant TB is endemic.^4^–^5^ Several studies are currently underway to search for new drugs reducing length of TB therapy to 3–4 months or less, and some new molecules [Bedaquiline (TMC-207), Delamanid (OPC-67683), PA-824 and others] or antibiotics used for other infections [*e.g.* moxifloxacin (MXF), gatifloxacin (GTF), rifapentine (RPT), linezolid (LZ), clofazimine (CFZ)], are currently being evaluated in appropriate combinations in phases II or III clinical trials or in observational studies.^2^–^3^ Vaccine development is also in progress, with at least 13 preparations being evaluated in phases I or II clinical trials.^2^,^6^ Currently, the only vaccine in use is the Bacillus Calmette-Guérin (BCG) vaccine, which protects children in the first 5–10 years of life especially against miliary and meningeal TB, but that does protect adults neither from pulmonary TB nor from reactivation of latent TB to active TB.^2^,^6^ Indeed, besides active TB about 2 billion people (one third of humanity) are estimated to be latently infected with Mtb, *i.e.* they harbor this organism in a nonreplicating (NR) (dormant) persistent stage somewhere in their tissues, as revealed by positivity to a skin test performed with a protein extract of Mtb known as tuberculin or purified protein derivative (PPD).^7^–^9^ It is estimated that 5% of tuberculin skin test (TST)-positive people develop pulmonary TB within 2 years after infection and that another 5% becomes ill lifetime for reactivation of dormant Mtb to an actively replicating (AR) stage. Because of the huge reservoir of latently infected individuals, it is important to know more about the biology of dormant Mtb in order to develop new therapeutic tools for active and latent TB with the ultimate goal of eradicating the tubercle bacillus from human beings.^2^,^7^–^8^,^10^

## Pathogenesis of TB

Although a single Mtb cell is potentially sufficient to infect a person, it is likely that only prolonged exposure to aerosols (1–10 μm in diameter) produced during coughing by patients with pulmonary TB causes transmission of the organism from sick people to healthy contacts. By microscopic observation of the sputum of the TB-patients using the Ziehl-Neelsen stain, also known as the acid-fast stain, Mtb cells are visualized as red bacilli, 3–4 μm in length. TB can affect any organ but in most cases (~ 80%) typically attacks the lungs. Mtb is transmitted through the air, and if it reaches pulmonary alveoli it is phagocytosed by macrophages and transported into the lung parenchyma where other cells are recalled to form granulomas, the hallmark tissue reaction of TB.^7^–^8^,^10^ These lesions contain activated macrophages and multinucleated giant cells surrounded by a rim of lymphocytes and by a fibrous capsule to circumscribe the field of battle between the immune system and Mtb. Different granulomas types (cellular, necrotic, caseous) may coexist within the same individual during active TB, due to different maturation stages of the lesion.^8^,^10^ The centre of the caseous granulomas, found in active and latent TB, is formed by a hypoxic and necrotic material (caseum) likely consisting of dead macrophages and other cells.^8^,^10^ Analysis of the lipid composition of caseum showed that it contains cholesterol, cholesteryl esters, triacylglycerols and lactosylceramide, and that development of the human TB granuloma to caseation correlated with pathogen-mediated disregulation of host lipid metabolism.^11^

Primary granulomas are formed mainly at the base of lungs (primary TB) and are caused by very low doses of infection (1–5 tubercle bacilli). In most of primarily infected people lesions resolve spontaneously with no symptoms while in the remaining 5–10% (more often children) local or systemic disease (menigeal or even miliary TB) develop within 1–2 years.^7^,^12^ In 90–95% of primary cases, Mtb infection evolves in latent infection with no symptoms; the TST become positive after 3–8 weeks and positivity is maintained throughout the entire lifetime likely due to persistence of NR Mtb in the tissues for many years. Mtb can also migrate via the lymphatics and the bloodstream from primary lesions to secondary sites located at the apical zones of the lungs with formation of post-primary granulomas (post-primary TB). For unknown reasons in about 10% of post-primary cases the immune system is unable to control the infection, allowing dormant bacilli to reactivate by multiplying to high density and increasing their concentration in the granulomas of the apical zones of the lungs. It is assumed that interaction with high amounts of Mtb antigens activates the immune response leading to the occurrence of caseous necrosis, liquefaction, cavity formation and release of the tubercle bacilli into airways of highly contagious pulmonary TB patients.^7^,^12^ Thus, the infection-disease-infection cycle mediated by reactivation of dormant Mtb in about 10% of TST-positive people with latent TB is the mechanism by which Mtb perpetuates its survival. In humans, a minimal size of around 0.1 mm^3^ was found to be essential for the initial formation of central necrosis.^13^–^14^ In patients with tuberculomas with latent TB the distant parts of lung tissue showed strong vascularization and proliferative activity, which lacked in cavitary TB lung lesions. Immune insulation of caseous granulomas may favor caseum liquefaction and cavity formation.^13^–^15^

## Dormant Mtb

In the hypoxic core of poorly vascularized necrotic and/or caseous granulomas the low oxygen pressure restricts the growth of aerobic AR Mtb to microaerophilic/anaerobic NR Mtb allowing bacilli to transit into a dormant state, *i.e.* a condition characterized by low metabolic activity that renders the bacteria resistant to killing by host immune response and antibiotics.

Initial response to hypoxia is regulated by the two-component response regulator DosR which, after phosphorylation by either of two sensor kinases (DosT, a hypoxia sensor, and DosS, a redox sensor) leads to induction of a set of 48 genes.^16^ Besides hypoxia, the DosR regulon is also induced in response to nitric oxide, nutrient starvation, and following infection of macrophages, mice, and guinea pigs. DosR regulon induction is transient, with about half genes returning to baseline within 24 hours. A second wave of gene expression then occurs after the initial DosR-mediated hypoxic response, consisting of 230 genes (Enduring Hypoxic Response, EHR) involved in the control of the regulatory factors and enzymatic machines of the long-term bacteriostasis program of NR Mtb.^17^

**Figure 1:**
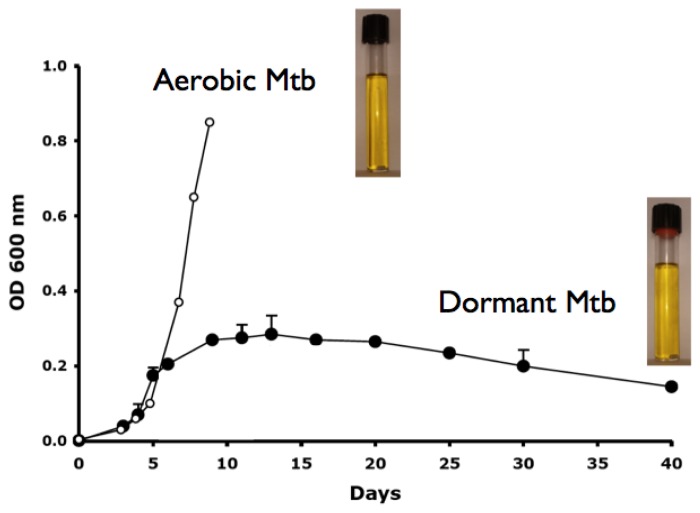
*Mycobacterium tuberculosis* (Mtb) grown for 40 days in 20- by 125-mm screw-cap tubes containing Dubos Tween-albumin broth, and stirred with 8-mm magnetic bars.^18^–^19^ Aerobic, replicating Mtb, was obtained by incubation of the tubes at 37°C with loosened screw caps. For preparation of dormant, nonreplicating bacilli, tight-fitting red rubber caps were put under the screw caps. Means and standard deviations of optical density (OD) at 600 nm are shown.

Several *in vitro* models to obtain NR Mtb *in vitro* have been developed over the years, based on reduced oxygen availability, nutrient starvation or the use of standing cultures. One of the most popular way is the Wayne model of hypoxia (Figure 1)^18^–^19^ in which dormant bacilli are obtained by gradual adaptation of stirred cultures of aerobic Mtb to anaerobiosis through the self-generated formation of an oxygen gradient. In this model at least two stages of nonreplicating persistence designated NRP-1 and NRP-2 occurs, with 1% (microaerophilic stage) and 0.06% (anaerobic stage) dissolved oxygen levels, respectively. Dormancy models including combined stresses of low oxygen, high CO_2_, low nutrients and acidic pH were also described.^20^ All these models are useful to screen drugs/drug combinations that can kill NR Mtb.

Dormant Mtb cells collected from lung tissues can be difficult to grow in culture media. In old studies examining surgically removed specimens from closed cavities of lungs of drug-treated patients, acid-fast bacilli were observed on microscopic sections but no colony growth was seen in solid media during the normal 8 weeks of incubation.^21^ However, by prolonging the incubation of cultures up to 3 to 10 months it was possible to obtain Mtb colonies, demonstrating the existence of few slowly growing tubercle bacilli surviving after drug treatment. Results from open cavities were in startling contrast to those from closed cavities, showing bacillary growth within 8 weeks. Differences in bacterial growth were also reported in more recent investigations, showing that Mtb was always cultivable from active cavitary TB but not from nonprogressive tuberculomas of healthy patients.^22^ To evade host responses, stresses bacteria can enter various reversible NR states characterized by impaired culturability.^23^ The peptidoglycan structure plays an important role in the maintenance of bacterial dormancy and a variation in a specific cross-link occurs during stationary-phase adaptation of Mtb. Five resuscitation-promoting factors (Rpf) similar to lyzozyme and lytic transglycosylases and sharing sequences with the Rpf of *Micrococcus luteus* were described and investigated for their capacity to resuscitate dormant mycobacteria.^24^ These studies indicated that activation of dormant cells by Rpf requires peptidoglycan hydrolysis facilitating cell division and/or the release of products acting as anti-dormancy signals.

There is strong evidence that Mtb uses lipids as a major energy source for persistence in the host. Fatty acids are stored as triacylglycerol in seed oils of plants and in the adipose tissues of mammals for use as energy during dormancy/hybernation. Mtb uses host triacylglycerol to accumulate lipid droplets intracellularly, and acquires a dormancy-like phenotype in lipid-loaded macrophages.^25^ Wax exter synthesis and utilization of host cholesterol are also required for Mtb to enter dormancy.^26^–^27^ Interestingly, intracellular lipid inclusion bodies were also observed in several Mtb cells isolated from the sputum of TB patients, as expected from the possibility that after tuberculoma disintegration a mixture of AR and NR Mtb is released into the airways.^28^ Mtb can also enter within adipocytes, where it accumulates intracytoplasmic lipid inclusions for survival in a NR state.^29^ Given the wide distribution of the adipose tissue throughout the body, Mtb may persist inside this tissue for long periods of time. Overall, due to the close relationship between lipid metabolism and dormancy, and the ability of NR Mtb to survive in lipid-reach caseous granulomas,^11^ cure and eradication of TB by drugs will be difficult.

Where does dormant Mtb live exactly? After treatment of guinea pigs with the novel drug TMC-207, Mtb was almost completely eradicated from the tissues, but few acid-fast bacilli were found to be extracellular within the central core of caseous necrosis and surrounding acellular rim of the primary granulomas.^30^–^31^ This zone was hypoxic and morphologically similar to that described for human lung lesions. The acellular rim may then be a primary location of persisting Mtb after drug treatment, highlighting the importance of developing new drugs/drug combinations able to eradicate extracellular Mtb remaining within necrotic lesions. In contrast, in drug-treated mice a homogenous reduction in viable counts was observed, indicating that the mouse model, which fails to show significant necrosis and hypoxia in lung lesions, is not suitable for the study of hypoxic responses of Mtb.^30^–^31^ Other investigators confirmed that TB granulomas of guinea pigs, rabbits and nonhuman primates, but not mice, are hypoxic.^32^ Indeed, measurements of pO_2_ in granulomas of Mtb-infected rabbits using pimonidazole labeling and a fiber optic oxygen probe revealed pO_2_ values lower than 2 mm Hg, in comparison with 151 mm Hg in the air and 37 mm Hg in murine Mtb lesions.^15^,^32^

The low efficiency of TB therapy is due to different reasons, including slow growth of Mtb related to periods of dormancy, low penetration of drugs in different granulomatous lesions, generation of drug-tolerant dormant persisters. In recent investigations Dartois et al compared by high-pressure liquid chromatography the distribution of INH, RIF, PZA and MXF in pulmonary lesions and plasma of Mtb-infected rabbits and found that MXF showed the best partitioning into lung and granulomas.^33^ However, more sophisticated measurements performed by MALDI mass spectrometry imaging showed that MXF and PA-824 do not diffuse effectively through caseum.^15^,^34^ Preliminary studies of this group suggested that PZA preferentially accumulates in caseum versus inflammatory cells.^15^

The prolonged treatment necessary to cure TB is also due to drug-tolerant dormant persisters *i.e.* a subpopulation of NR or slowly replicating bacilli that survive the cidal action of antibiotics.^35^ These cells have noninheritable phenotypic resistance or tolerance to antibiotics, but their progeny is fully susceptible to drugs. Mtb persisters likely comprise different subpopulations and consist of a very small proportion of Mtb. To give an example *in vivo*, in the lungs of guinea pigs treated for 6 weeks with 15 mg/kg of TMC-207, 1 persisting Mtb out of about 20,000 bacilli was recovered after treatment.^30^*In vitro,* low numbers of Mtb persisters are present in early exponential phase, but their number increases sharply up to 1% of the population at late exponential and stationary phases.^36^ Thus, their proportion likely depends by specific conditions including the age of a culture, the length of drug exposure, the type and concentration of antibiotics.^35^ It is not yet clear while their formation can be promoted in a stochastic or deterministic (for instance by induction) manner but it is possible that both mechanisms are involved.^35^–^37^ A study of the transcriptome of Mtb persisters identified a small number of genes upregulated by different stresses [antibiotic exposure, increasing hypoxia (Wayne model), hypoxia by continuous flow of low oxygen (EHR), nutrient starvation in phosphate buffered saline] which may represent a core dormancy response.^36^ Recently it was reported that mycobacterial persisters may be eradicated *in vitro* with antibiotic-generated hydroxyl radicals, suggesting that stimulation of reactive oxygen species provides a potential strategy to managing persistent infections.^38^ This observation is in keeping with the knowledge that bactericidal antibiotics may stimulate the production of highly deleterious hydroxyl radicals in Gram-negative and Gram-positive bacteria.^39^

## Activity of Drugs Against Dormant Mtb

The most important objective for discovery of new anti-TB drugs is to find new drugs/drug combinations able to effectively eradicate *in vivo* both AR (aerobic) bacilli and NR (microaerophilic, anaerobic and persisters) bacilli to shorten therapy of active TB below 6 months, and effectively reduce the reservoir of latently infected individuals. Using the Wayne model and other models it was shown that NR bacilli were insensitive to INH, while being inhibited by RIF, PZA, fluoroquinolones (*e.g.* MXF), aminoglycosides [*e.g.* amikacin (AK)], capreomycin (CP) and nitrocompounds [*e.g.* metronidazole (MZ), niclosamide (NC), nitazoxanide (NTZ), PA-824 and other drugs].^3^,^19^,^35^ Among nitrocompounds, MZ strongly reduced Mtb viable counts under anaerobic conditions, but showed no activity under aerobic conditions.^3^,^18^ Using the Wayne model we found that the combination MZ+RIF sterilized long-term (26-days-old) dormant Mtb cultures and that some combinations (RIF+MXF+MZ+AK or RIF+MXF+MZ+CP) killed both AR and NR Mtb, as measured by a test of residual viability more sensitive than colony forming units (CFU) based on the lack of re-growth after 100 days of incubation in liquid medium (MGIT 960).^40^–^41^*In vivo* activity of MZ was reported to be different in various animal models, and was related to the presence of caseous necrosis, or to drug toxicity. Indeed, MZ had no activity in mice and was toxic in a combination treatment in guinea pigs but showed good efficacy in rabbits and prevented reactivation of latent TB in macaques.^42^ When administered to MDR-TB patients in a controlled trial, MZ increased early sputum smear and culture conversion, but was too neurotoxic to use over the long term.^43^ Due to MZ toxicity, novel and better tolerated nitroimidazoles with activity against both aerobic and anaerobic Mtb are presently investigated *in vitro* and *in vivo* with promising results, indicating the importance of including at least a drug with anaerobic activity in newly designed regimens. The novel nitroimidazole PA-824 demonstrated anti-TB activity in combination with other drugs in mice and in human early bactericidal activity (EBA) studies.^43^ The drug inhibits the mycolic acid biosynthesis of aerobic Mtb and kills NR Mtb by intracellular nitric oxide release.^3^,^44^ In the Wayne model we showed that the combination (RIF+MXF+AK+PA-824) was more efficient than the combination currently used in human therapy (RIF+INH+PZA+EMB) and killed both AR and NR Mtb in 14 days.^19^ Also TMC-207, a diarylquinoline recently approved with restraint by the Food and Drug Administration for treatment of MDR-TB, is effective against both AR and NR Mtb.^45^–^47^ The drug acts specifically by targeting the membrane-bound c-subunit of ATP synthase of AR Mtb and the residual ATP synthase enzymatic activity of NR Mtb.

Since anti-TB treatment requires activity of drugs against both AR and NR bacilli living in a wide spectrum of lung lesions, use of drug combinations is essential to cover all of these requirements. Understanding where a bacterial population resides and evaluating the ability of novel molecules to penetrate a site allows a more rational approach to the design of new combinations. Traditional Minimum Inhibitory Concentration (MIC) measures the activity of a compound only against AR aerobic cells, representing a non-physiological situation in low or no vascularized necrotic (caseous) granulomas.^15^ Thus, NR or slowly replicating Mtb assays have been developed including low oxygen (Wayne model), nutrient starvation and nitric-oxide release assays using MZ and INH as positive (growth) and negative (no growth) controls, respectively.^48^ The necrotic tissue has a pH of about 6.5^49^ while the pH of active TB lesions is between 5.5 and 6.0 or lower.^19^ The standard Wayne model of hypoxia at pH 6.6 or the modified version at pH 5.8 we recently set up^18^–^19^,^40^–^41^ may mimic the microenvironment of necrotic and cellular granulomas, respectively, and be suitable for measuring the activity of new compounds under different hypoxic conditions. To eradicate Mtb from the tissues we need to kill both AR and NR Mtb that are not eliminated with current therapy. A complication in the discovery of new drugs/combinations is the lack of a sensitive test determining when Mtb is dead.^8^ Considering that NR Mtb may not form colonies on agar, we found that re-growth of drug-exposed Mtb in a liquid medium (MGIT 960) was a test much more sensitive than CFU measurements to demonstrate the ‘cidal’ activity of a combination.^19^,^41^ We think that this test should be used together with the CFUs when studying the sterilizing activity of new drugs or combinations.

Besides *in vitro* tests the sterilizing activity of a combination against AR and NR Mtb can be determined in animal models reaching different stages of granuloma formation ranging from mouse model (lacking organized granulomatous structures) to guinea pig, rabbit and macaque models, showing necrosis, caseation, liquefaction and cavity formation.^15^ No one animal model fully mimics the complete spectrum of lesions shown by humans. However, in the practice, the most popular model for determining the sterilizing activity of drug combinations is still the mouse model which, due to low costs and drug requirements, is widely used by monitoring CFUs in organs and the relapse rate 3 months after discontinuation of treatment. Recently, some combinations sterilizing Mtb-infected mice were reported, including TMC-207+PZA+CFZ+RPT (6 weeks), TMC-207+PZA+CFZ (8 weeks), TMC-207+PZA+RPT (8 weeks).^50^–^51^ However, due to lack of hypoxia and necrosis in mouse lungs, efficacy of single drugs and combinations not necessarily predicts the clinical feature in humans.^15^ Interestingly, the use of a hypoxic mouse model (C3HeB/FeJ mice) developing lesions with liquefactive necrosis is under validation for testing new drugs and combinations.^52^ In the guinea pigs model, the combination PA-824+MXF+PZA killed Mtb from lungs in 8 weeks and showed the highest EBA values in humans.^53^–^54^ This and at least other 37 drug combinations containing high-doses INH, RIF, RPT or fluoroquinolones, TMC-207, PA-824, OPC-67683, and other drugs (SQ-109, PNU-100480, AZD-5847) are in Phases I, II or III clinical trials against drug-susceptible and -resistant TB, and results are expected to be reported in the next few years.^2^–^3^
